# Biocatalytic Indigo Synthesis From L‐Tryptophan Using a Three‐Step Cascade Without Cofactor Regeneration

**DOI:** 10.1002/cbic.70342

**Published:** 2026-04-21

**Authors:** Vivian P. Willers, Nikola Lončar, Marco W. Fraaije

**Affiliations:** ^1^ Molecular Enzymology University of Groningen Groningen The Netherlands; ^2^ GECCO Biotech Groningen The Netherlands

**Keywords:** biocatalysis, enzyme cascade, indigo, L‐tryptophan, peroxygenase

## Abstract

Indigo is currently produced from petrochemical sources, which poses significant environmental challenges. Sustainable biotechnological alternatives are therefore highly desirable. Enzymatic synthesis of indigo from L‐tryptophan via indole has been demonstrated, but conventional pathways based on flavin‐containing monooxygenases require costly coenzymes such as NAD(P)H, limiting their practical applicability. In this study, we present a novel, self‐sufficient, NAD(P)H‐independent enzyme cascade for indigo biosynthesis from the renewable feedstock L‐tryptophan. The cascade starts with conversion of L‐tryptophan into indole and pyruvate by a tryptophanase. As next steps, the system couples an engineered bacterial tyrosine hydroxylase, which converts indole into indoxyl using hydrogen peroxide, with a pyruvate oxidase that generates the required peroxide in situ. The cascade thereby transforms a reaction byproduct into the oxidizing equivalent needed for the subsequent step, establishing a closed catalytic cycle with minimal auxiliary inputs. After optimizing cascade parameters, the system produced 0.25 mM indigo from 5 mM L‐tryptophan. Although the overall yield remains moderate, this proof‐of‐principle demonstrates a sustainable and cost‐effective enzymatic route for indigo production from biobased starting materials, providing an environmentally friendly alternative to petrochemical synthesis.

## Graphical abstract

A self‐sufficient, NAD(P)H‐independent enzyme cascade for the synthesis of indigo from L‐tryptophan. The minimal cascade uses pyruvate, a byproduct of tryptophan cleavage, to generate hydrogen peroxide, which fuels the key hydroxylation of indole to indoxyl by an engineered tyrosine hydroxylase. Indoxyl then spontaneously dimerizes under aerobic conditions to form indigo, creating a closed‐loop system for sustainable production from renewable resources.

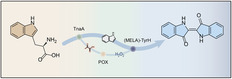

## 1 Introduction

Dyes and pigments have played a vital role in human history, serving both esthetic and functional purposes for millennia. Among the most renowned and ancient dyes is indigo, with records of its use dating back as far as 4000 B.C [1]. A precursor of indigo is found naturally in plants of the *Indigofera* genus, where it is present as the glycoside indican [2]*.* Traditional extraction methods involved hydrolysis of indican to indoxyl, which then spontaneously oxidizes in air to form the deep blue indigo. The resulting pigment could then be harvested and processed into a stable dye powder [3]. With the advent of industrial chemistry, natural extraction methods were replaced by synthetic production. Today, indigo is manufactured on a large scale using petrochemical precursors such as aniline or nitrobenzene [4, 5]. Although efficient, these synthetic routes involve harsh conditions, high energy consumption, and the generation of toxic waste streams, raising concerns about sustainability and environmental impact [6].

In response, biotechnological approaches have gained increasing attention as greener alternatives for indigo production. Early efforts demonstrated microbial pathways combining tryptophanase and naphthalene dioxygenase, enabling the conversion of glucose to indigo in *Escherichia coli* with titers reaching up to 18 g L^−1^ [7, 8]. Since then, various hydroxylating enzymes harboring flavin, heme or non heme‐iron cofactors have been explored for the conversion of indole to indoxyl, the key intermediate in indigo formation [4]. Currently, most explored enzymatic routes to indigo involve flavin‐containing monooxygenases (FMOs). These flavoenzymes catalyze the NAD(P)H‐dependent hydroxylation of indole to indoxyl. Enzyme cascades involving FMOs have achieved titers up to 2 g L^−1^ from L‐tryptophan [9]. However, their dependence on the expensive coenzyme NAD(P)H presents a significant limitation for large‐scale applications. Although coenzyme recycling systems have been introduced, they must be highly efficient to achieve a cost‐effective process. Furthermore, the microbial production of indigo is constrained by the cytotoxicity of indole, which inhibits bacterial growth at low millimolar concentrations [10]. This further underscores the need for efficient, robust, and coenzyme‐independent enzymatic systems.

To address the limitations of NAD(P)H dependence, unspecific peroxygenases (UPOs) have emerged as a promising alternative. UPOs are secreted fungal heme‐thiolate enzymes that catalyze the peroxide‐driven hydroxylation of a wide range of substrates, including indole, without requiring NAD(P)H. Their ability to perform oxygen transfer reactions using only hydrogen peroxide makes them attractive for sustainable biocatalysis. However, their broader application is limited by several challenges. These include difficulties in heterologous expression, particularly in bacterial systems, and susceptibility to inactivation by excess hydrogen peroxide. Moreover, their use in enzyme engineering is constrained by limited expression yields and a lack of high‐throughput screening systems. Despite their excellent catalytic potential, these factors hinder their implementation and optimization in scalable production [11, 12].

Building on these limitations, Carraretto et al. previously engineered a heme‐dependent tyrosine hydroxylase from the bacterium *Streptomyces sclerotialus* (ssTyrH) capable of catalyzing peroxygenase‐like hydroxylation reactions on substrates such as indole and thioanisole. In contrast to fungal UPOs, ssTyrH and variants thereof can be efficiently expressed in *E. coli*, offering a more practical route for its engineering and use in various applications [13]. In the present study, we build upon this work by establishing a fully enzymatic, NAD(P)H‐independent cascade for the biosynthesis of indigo from L‐tryptophan. To drive the peroxygenase reaction, we incorporated a recently characterized pyruvate oxidase [14] that converts pyruvate, a side product of tryptophan cleavage, into acetyl phosphate and hydrogen peroxide, thus supplying the required oxidant in situ. This conceptually novel, coenzyme‐free three‐step cascade enables NAD(P)H‐free indigo formation with a minimum set of enzymes and additives adding a novel biocatalytic strategy to the toolbox of sustainable indigoid dye biosynthesis.

## 2 Results and Discussion

### 2.1 Concept of In Vitro Biocatalytic Cascade for the Coenzyme‐Free Production of Indigo

To establish an efficient in vitro route toward indigo, we designed a NAD(P)H‐independent enzymatic cascade centered on the recently engineered bacterial tyrosine hydroxylase variant (MELA‐TyrH), which catalyzes the hydroxylation of indole using hydrogen peroxide as the sole oxidant. In contrast to classical monooxygenase‐based systems, this approach eliminates the need for auxiliary cofactor recycling [9]. Furthermore, MELA‐TyrH can be efficiently expressed in *Escherichia coli*, unlike fungal UPOs, which frequently exhibit limited expression levels and operational stability. This accessibility, combined with their tunability through protein engineering, renders these enzymes particularly suitable for the development of tailored peroxygenase‐type cascades. However, peroxygenase‐type reactions inherently depend on the controlled supply of stoichiometric H_2_O_2_, as excess oxidant rapidly leads to enzyme inactivation [15, 16]. To address this challenge, we envisioned a self‐sufficient, closed‐loop cascade in which L‐tryptophan not only provides the substrate (indole) but simultaneously fuels in situ oxidant generation. Tryptophanase (ecTnaA) cleaves L‐tryptophan into indole and pyruvate in equimolar amounts. While indole enters the hydroxylation pathway, we repurposed the side product pyruvate to drive controlled H_2_O_2_ formation. To this end, we incorporated a pyruvate oxidase from *Aerococcus viridans* (avPOX), which efficiently converts pyruvate and phosphate into acetyl phosphate while forming H_2_O_2_ from molecular oxygen [14, 17]. Importantly, in situ H_2_O_2_ generation has been successfully employed in related biocatalytic systems, where it enables tighter oxidant control and improved enzyme stability compared to continuous external dosing [18, 19, 20].

**FIGURE 1:**
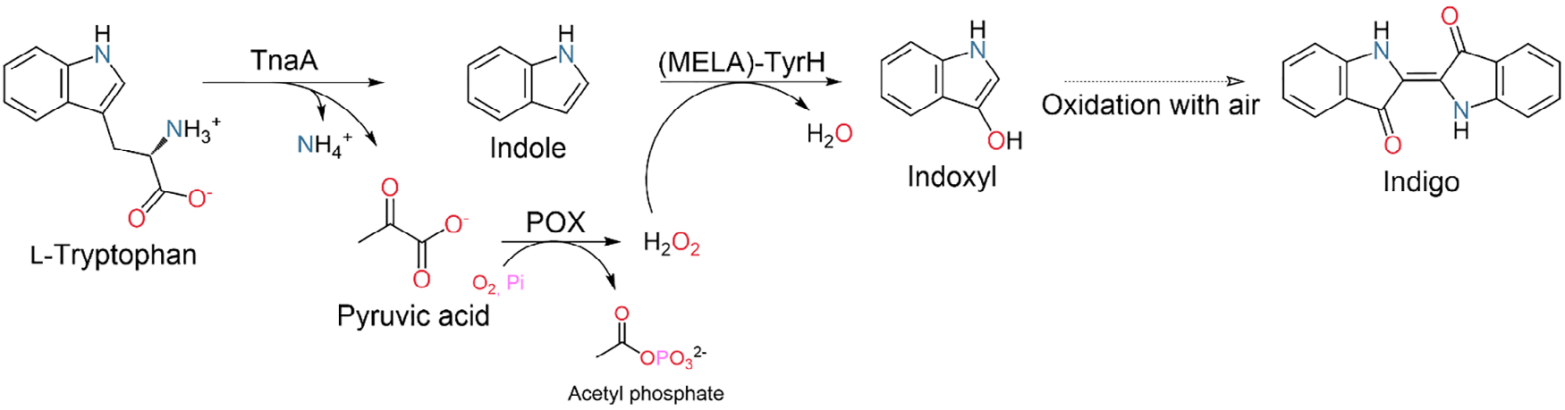
Enzyme cascade for the synthesis of indigo from L‐tryptophan (TnaA: tryptophanase, POX: pyruvate oxidase, MELA‐TyrH: quadruple variant of ssTyrH).

The resulting three‐enzyme cascade, ecTnaA, avPOX, and MELA‐TyrH (Table S1), thus establishes a minimal, coenzyme‐free and self‐sufficient biocatalytic route to indigo (Figure 1). In contrast to in vivo systems, the simplicity of the in vitro system and the water‐insolubility of indigo represent practical advantages over whole‐cell approaches, where cytoplasmic accumulation complicates product recovery [5].

### 2.2 Enzyme Characterization and Pathway Optimization

To establish the proof‐of‐principle cascade, each required enzyme (ecTnaA, avPOX, and MELA‐TyrH) was heterologously expressed in *E. coli* (Table S1)*.* ecTnaA was produced with an N‐terminal His_6_‐tag, whereas avPOX and MELA‐TyrH were expressed as Small Ubiquitin‐like Modifier protein fusions (SUMO‐fusions) carrying an N‐terminal His_6_‐tag on the SUMO domain. All proteins were purified by nickel affinity chromatography. The expression and purification protocols resulted in excellent yields per liter of culture: 230 mg ecTnaA, 120 mg avPOX, and 200 mg MELA‐TyrH. The catalytic properties of ecTnaA and avPOX were first determined under standard reaction conditions (Table 1, Figure S1). ecTnaA and avPOX exhibited turnover numbers (k_cat_) of 0.7 s^−1^ on L‐tryptophan and 3.2 s^−1^ on pyruvate, respectively. MELA‐TyrH displayed the lowest k_cat_ (0.29 s^−1^) using indole as substrate [13]. The Michaelis–Menten constants (K_M_) for avPOX and MELA‐TyrH were 3.8 mM for pyruvate and 1.4 mM for indole, respectively. Enzyme stability was evaluated in terms of thermal and operational robustness (Table 1, Figure S2). ecTnaA and MELA‐TyrH exhibited apparent half‐lives (t_1/2_) exceeding 24 h at 25°C. avPOX, however, displayed a rapid initial activity loss of ∼50% within 21 min, after which a stable residual activity remained, suggesting partial inactivation. Short‐term thermal challenges revealed T_50_ values of 58°C for ecTnaA, 32°C for avPOX, and 41°C for MELA‐TyrH.

**TABLE 1:** Enzyme parameters including kinetic properties as well as thermal and operational robustness.

Enzyme	T_50_, °C	t_1/2_, 25°C	k_cat_, s^−1^	K_M_, mM	Reference
ecTnaA	57	>24 h^a^	0.71 ± 0.03	0.16 ± 0.03	This study
avPOX	32	21 min^b^	3.20 ± 0.01	3.79 ± 0.08	This study
MELA‐TyrH	41	>24 h^a^	0.29^c^	1.37^c^	c[13]

a For ecTnaA and MELA‐TyrH, activity loss did not follow first‐order kinetics; the apparent half‐life exceeded 24 h.

b Data were fitted to a two‐phase (bi‐exponential) decay; the slower phase corresponded to a half‐life of approximately 27 h.

c Values taken from Ref. [13].

Taken together, these data reveal complementary yet unbalanced catalytic features of the three biocatalysts. While ecTnaA is both catalytically and thermally robust, avPOX combines high activity with pronounced thermal sensitivity, and MELA‐TyrH displays moderate stability but comparatively low efficiency. Especially, the high Michaelis‐Menten constant for hydrogen peroxide of MELA‐TyrH presents a dual challenge: while a certain concentration of H_2_O_2_ is required to activate the enzyme for indole conversion, excessive peroxide can inactivate MELA‐TyrH and other cascade enzymes. This tradeoff underscores the need for precise control of oxidant generation to preserve activity and maintain turnover.

### 2.3 Optimization of Enzyme Ratios for Efficient Indigo Formation

**FIGURE 2:**
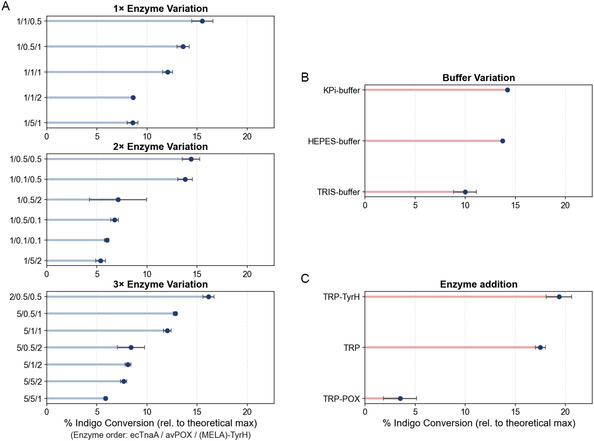
Initial cascade optimization with parameter variation on the *y*‐axis and produced indigo after 20 h of incubation normalized to the theoretical yield derivable from 1.0 mM L‐tryptophan on the *x*‐axis. (A) Enzyme variations, with increase or decrease of the concentration of one, two or all three cascade enzymes (enzyme order: ecTnaA / avPOX / MELA‐TyrH). 1x enzyme is normalized to 0.1 U mL^−1^. (B) Additional variations of buffer (enzyme ratio (1x /0.5x /1x), as well as temporal control of enzyme addition. For the temporal control of enzyme addition, the *y*‐axis shows initial enzymes added at the start of the cascade, missing enzyme(s) were added after 3 h of incubation (final ratio 1x /0.5x /0.5x).

Initial attempts to normalize enzyme levels based solely on protein concentration (1:1:1 ratio) failed to yield detectable indigo (data not shown), indicating that equal protein amounts do not ensure balanced activity in multienzyme cascades. To address this, enzyme concentrations were instead normalized based on activity (U mL^−1^ based on initial activity measurements). Each enzyme was adjusted to 0.1 U mL^−1^ in 50 mM potassium phosphate buffer (pH 8.0). By switching from concentration‐ to activity‐based normalization, we observed the formation of a blue color in the reaction mixture, which over time led to precipitation. After centrifugation and dilution in DMSO, the characteristic absorbance spectrum of indigo was obtained. Encouraged by the observed product formation, we subsequently varied individual enzyme concentrations by factors ranging from 0.1× to 5×. Reactions were incubated for 20 h, and indigo formation was quantified (Figure 2). Indigo production proved highly sensitive to enzyme ratios. Both overrepresentation (5×) and underrepresentation (0.1×) of avPOX led to decreased yields, likely due to imbalances in H_2_O_2_ generation. Similarly, nonoptimal levels of MELA‐TyrH reduced conversion efficiency despite adequate indole and H_2_O_2_ supply (Figure S3). The highest yields were obtained under conditions where avPOX was maintained at 0.1 U mL^−1^ or reduced to 0.05 U mL^−1^, with MELA‐TyrH also reduced to 0.05 U mL^−1^, while ecTnaA was increased to 0.2 U mL^−1^. Under these optimized conditions, indigo yield reached approximately 15% of the theoretical maximum based on the initial tryptophan concentration (1 mM). Counterintuitively, increasing MELA‐TyrH while decreasing avPOX suppressed indigo formation. This may be due to overoxidation of reactive intermediates or the generation of nonproductive side products such as 2‐oxindole (Figure S4).

### 2.4 Buffer Effects on Cascade Performance

To assess how buffer composition influences cascade performance, we tested three systems at pH 8.0 over a 20‐h reaction period, using enzyme ratio of 0.5× avPOX, 1× MELA‐TyrH, and 1× ecTnaA. The buffers evaluated were: (i) 50 mM potassium phosphate (KPi), (ii) 50 mM HEPES supplemented with 5 mM KPi, and (iii) 50 mM TRIS supplemented with 5 mM KPi. Addition of some KPi was needed to provide the avPOX with the required phosphate. Both the KPi and HEPES‐based buffers supported high indigo yields, whereas the TRIS‐containing system led to a marked decrease in product formation (Figure 2B). Supplementation of 50 mM KPi to the HEPES and TRIS systems did not significantly improve conversion (Figure S5), indicating that phosphate availability is not the limiting factor. Instead, the reduced performance is attributed to the buffer, as TRIS is known to exhibit metal‐chelating properties and has been reported to negatively affect PLP dependent enzymes and tryptophanase activity [21, 22] .

### 2.5 Temporal Control of Enzyme Addition

To further optimize cascade efficiency, we investigated the effect of temporal coordination of enzyme addition using the previously identified optimized enzyme ratio (1× ecTnaA, 0.5× avPOX, 0.5× MELA‐TyrH). In alternative setups, ecTnaA, ecTnaA and avPOX or ecTnaA and MELA‐TyrH, was added at time zero, while avPOX and/or MELA‐TyrH were introduced after a 3‐h delay (Figure 2C). Delayed addition of avPOX after 3 h resulted in a slight increase in indigo yield compared to the simultaneous condition (19% indigo of the theoretical maximum based on starting tryptophan concentration), likely due to reduced early accumulation of H_2_O_2_ and improved preservation of downstream enzyme activity. A slight improvement was observed when avPOX and MELA‐TyrH was delayed, indicating that managing the timing of peroxide production is a key factor in maintaining cascade performance. In contrast, delaying only the addition of MELA‐TyrH while adding avPOX together with ecTnaA at the start resulted in a marked reduction in indigo formation. In this case, nearly no indole was converted further (Figure S3) and only a low amount of 2‐oxindole was observed (Figure S4). This suggests that early peroxide production by avPOX, in the absence of a coupled downstream reaction, may lead to the accumulation of H_2_O_2_ beyond tolerable levels. Such accumulation likely causes oxidative inactivation of one or more enzymes in the cascade. Peroxygenases are particularly susceptible to inactivation by hydrogen peroxide. For MELA‐TyrH, the addition of H_2_O_2_ led to a concentration‐dependent loss of activity, with 2.5 mM reducing activity by ∼20% and 10 mM by nearly 50% (Figure S6A). Consistently, MELA‐TyrH has been reported to lose approximately 50% of its activity within 10 min in the presence of 1 mM H_2_O_2_. In contrast, tryptophanse did not exhibit a pronounced decrease in activity under comparable conditions (Figure S6B). These findings indicate that early accumulation of H_2_O_2_ primarily compromises MELA‐TyrH, highlighting the importance of controlled oxidant supply for maintaining cascade performance.

### 2.6 Indigo Production from L‐Tryptophan

**FIGURE 3:**
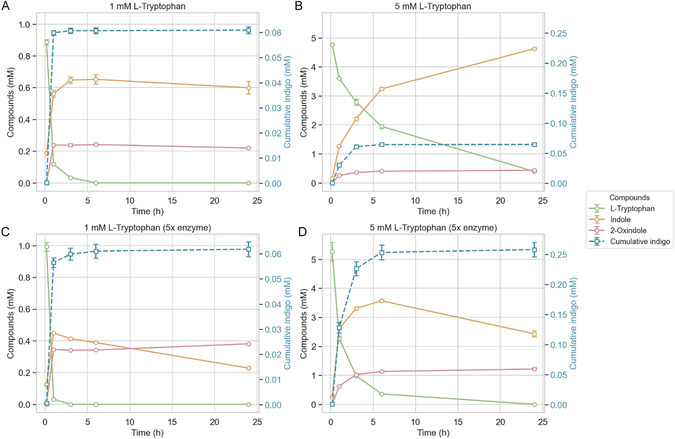
Time course experiments for the conversion of L‐tryptophan (1.0 or 5.0 mM) using a three‐enzyme cascade over 24 h. At each time point, indigo was removed from the reaction mixture by centrifugation and quantified. Because indigo was physically removed at each step, its concentration in the mixture did not accumulate. Therefore, cumulative indigo concentrations were calculated by summing the amounts measured across all time points (e.g., cumulative at 3 h = 0 h + 1 h + 3 h). (A) Conversion of 1.0 mM L‐tryptophan using the optimized enzyme ratio (1× ecTnaA /0.5× avPOX /0.5× MELA‐TyrH). (B) Conversion of 5.0 mM L‐tryptophan using the optimized enzyme ratio (1×/0.5×/0.5×). (C) Conversion of 1.0 mM L‐tryptophan using an increased enzyme dosing (5×/2.5×/2.5×). (D) Conversion of 5.0 mM L‐tryptophan using an increased enzyme dosing (5×/2.5×/2.5×).

The endpoint measurements alone provided limited insight into cascade performance and did not allow further optimization. We performed time‐course experiments to track substrate consumption, intermediate accumulation, and byproduct formation over 24 h (Figure 3, Figure S7). These reactions were conducted under optimized enzyme ratios, without temporal control of enzyme addition, using either 1 or 5 mM L‐tryptophan, in 1 mL total volume. Together with this, we supplemented FAD (100 µM) to the reaction as it was shown previously that it can have a stabilizing effect on the pyruvate oxidase of *Lactobacillus plantarum* [23]. Soluble metabolites were analyzed from the supernatant, while insoluble indigo was extracted from the pellet using DMSO and quantified at 620 nm.

At a starting concentration of 1.0 mM L‐tryptophan, complete substrate depletion was observed within 6 h. Indole accumulated concurrently, reaching a peak concentration of 0.65 mM at 6 h, followed by a slight decrease to 0.60 mM by 24 h. Indigo formation was restricted to the first 3 h, plateauing at approximately 0.06 mM. A major side product, 2‐oxindole, accumulated in parallel with indigo, reaching 0.24 mM at 6 h and decreasing slightly to 0.22 mM after 24 h.

A time‐course experiment was also conducted at a starting concentration of 5.0 mM L‐tryptophan. Substrate conversion was nearly complete after 24 h. Indole accumulated steadily, reaching 4.6 mM at 24 h. Despite this, indigo levels remained low (∼0.06 mM after 24 h), although its formation was observed over a longer period (up to 6 h), albeit at a slower rate. In this setup, 2‐oxindole levels increased gradually, reaching 0.4 mM by 24 h. These results suggest that a substantial fraction of indole was diverted toward formation of 2‐oxindole. These findings point to a bottleneck in selectivity of the peroxygenase and highlight the need for improved enzyme characteristics together with further cascade balancing. A likely scenario is that H_2_O_2_ is initially generated in excess, leading to partial oxidative inactivation of cascade enzymes; subsequent indigo formation then proceeds slowly through residual or partially active enzymes. L‐tryptophan, however, is converted completely over 24 h, indicating that tryptophanase remains stable, whereas other enzymes may lose activity before full substrate consumption.

To address the reduced conversion efficiency observed at higher substrate concentrations, we hypothesized that one of the enzymes, most likely avPOX, was inactivated during the early stages of the reaction. To mitigate this, we increased the concentration of all cascade enzymes fivefold and repeated the experiment (Figure 3C,D). In the 1.0 mM substrate setup, indigo production remained unchanged. However, in the 5.0 mM reaction, cumulative indigo levels increased approximately fourfold, reaching ∼0.25 mM, corresponding to a conversion efficiency comparable to that of the 1.0 mM system. This enhanced indigo formation was accompanied by improved tryptophan turnover and increased 2‐oxindole accumulation. Interestingly, while indole levels began to decline after 6 h, indigo formation had already ceased by that time. By 24 h, a pink coloration developed in the reaction mixture (Figure S8), potentially indicating the formation of indirubin as a side product. This would be in agreement with the reduction in indole concentration after 6 h. The increase in enzyme concentrations improved cascade efficiency in the 5.0 mM setup, suggesting that rapid enzyme inactivation occurred at lower enzyme loadings. These data also show that early accumulation of indole supports higher indigo titers. This conclusion aligns with the temporal control experiment, where indole was first generated before addition of avPOX to supply H_2_O_2_. Under these conditions, indole levels were initially high while hydrogen peroxide gradually increased, maintaining a favorable ratio for selective hydroxylation. However, increasing indole titers showed a inhibitory effect on tryptophanase [24]. Tryptophanase activity on 1 mM tryptophan was completely diminished when adding 5 mM indole to activity measurements (Figure S6C), which might be the reason why indole production declined over time. Indole had also an inactivating effect on pyruvate oxidase activity whereby a five times overshoot in indole to pyruvate decreased activity by 35%. By increasing pyruvate to the same level of indole, 24% inactivation was observed. Together, these results highlight a narrow operational window for the applied peroxygenase in which peroxide generation and substrate hydroxylation must be finely balanced for optimal cascade function. Comparable challenges have been reported for other indigo production systems, where precise regulation of oxidant levels is essential to maintain activity while avoiding catalyst damage. Liu et al. (2018) engineered a sperm whale myoglobin to catalyze the conversion of indole to indigo, finding that high indole titers (4 mM) combined with lower peroxide concentrations (1 mM) yielded the best results, whereas higher peroxide levels caused enzyme inactivation [25]. Similarly, in an iron(III)‐porphyrin system described by Rebelo et al., optimal conversion was achieved when approximately two molar equivalents of oxidant were titrated relative to substrate over time [26]. Another limitation of the current cascade lies in the regioselectivity of the employed peroxygenase. The cascade currently favors 2‐oxindole formation, suggesting that MELA‐TyrH predominantly hydroxylates indole at the C2‐position rather than the C3‐position required for indigo synthesis. Similar regioselectivity patterns have been observed for peroxide‐dependent enzymes such as chloroperoxidases and UPOs [27, 28, 29], likely reflecting intrinsic differences in active‐site architecture. Further mechanistic and engineering studies may enable tuning of MELA‐TyrH toward C3‐hydroxylation, resulting in improved indigo production.

## 3 Conclusion

In summary, this work establishes a proof of principle for a self‐sufficient, coenzyme‐independent enzyme cascade for indigo biosynthesis. The cascade uses a reaction byproduct, pyruvate, to form hydrogen peroxide required for the next oxidative step, creating a closed catalytic route with minimal auxiliary inputs. Indigo can be readily harvested by centrifugation, while the soluble cascade intermediates remain in the supernatant, which highlights a practical advantage of in vitro enzyme cascade systems. While the overall conversion of L‐tryptophan into indigo was somewhat limited, this design marks an important first step toward a minimal and self‐sufficient enzymatic framework for indigo biosynthesis. Beyond indigo, the approach provides a generalizable concept for sustainable biocatalytic oxidations in which substrate‐derived oxidants drive peroxygenase catalysis. Future improvements in enzyme stability and selectivity, kinetic balance, and peroxide management will be key to unlocking the potential of this system for scalable indigo production.

## 4 Experimental Section

Common chemicals and medium components were purchased from Sigma‐Aldrich, Merck or Thermo Fisher. Horseradish peroxidase (HRP), FAD, L‐arabinose, L‐tryptophan, indole, indigo, 2‐oxindole, pyridoxal phosphate and thiamin pyrophosphate were purchased from Sigma‐Aldrich. 5‐Aminolevulinic acid was purchased from Merck. Lactate dehydrogenase (LDH) was purchased from Roche. The gene encoding avPOX was purchased at Twist Bioscience as gene optimized for expression in *E. coli*, with flanking *BsaI* cutting sides. The PfuUltra Hotstart PCR master mix was purchased from Agilent Technologies. T4 ligase and the restriction enzyme *BsaI* were purchased from New England Biolabs. Chemically competent *E. coli* NEB 10‐beta cells (New England Biolabs) were used as cloning and expression strain.

### 4.1 Molecular Cloning and Plasmid Construction

The gene encoding avPOX was introduced into a pBAD‐SUMO expression vector through Golden Gate cloning. The Golden Gate reaction (total volume 20 µL) contained vector (75 ng), synthetic gene (synthetic gene: vector in a 3:1 molar ratio), *BsaI*‐HFv2 (New England Biolabs, UK) (15 U µL‐1), T4 DNA ligase (15 U µL‐1), and T4 DNA ligase buffer. The reaction started with incubation at 37°C (5 min), 16°C (10 min) for 30 cycles, 55°C (10 min), 65°C (20 min), and cooling down to 4°C (T100 PCR thermal cycler, Biorad). The final construct result in a N‐terminal fusion of the avPOX fused to a His_6_‐tagged SUMO protein. A pBAD‐SUMO vector containing the gene encoding MELA‐TyrH was provided by Carraretto et al. [13]. Similar to the avPOX‐encoding gene, the construct results in expression of MELA‐TyrH fused to a His_6_‐tagged SUMO protein. A pBAD vector containing the gene encoding tryptophanase (ecTnaA) of *E. coli* was prepared during a previous study [9]. The construct results in expression of the tryptophanase with an N‐terminal His_6_‐tag.

### 4.2 Protein Expression and Purification

For expression of recombinant proteins, a preculture was prepared in 10–20 mL of Lysogeny Broth (LB) medium containing 50 µg mL^−1^ ampicillin in a baffled shaking flask. The medium was inoculated with a colony from freshly transformed *E. coli* NEB 10‐beta cells from an LB‐Agar plate containing 50 µg mL^−1^ ampicillin. The culture was incubated for 16–20 h at 37°C with shaking at 200 rpm. The preculture was used to inoculate a main culture. For that, 400 mL Terrific broth (TB) medium containing 50 µg mL^−1^ ampicillin, in a 2 L baffled shaking flask, was inoculated to an optical density at 600 nm (OD_600_) of 0.05 and incubated at 37°C with 200 rpm shaking until an OD_600_ of 0.6–0.8. Heterologous protein expression was induced by addition of L‐arabinose to a concentration of 0.02%. After induction, the culture was incubated for 20 h at 24°C with 200 rpm. For the expression of MELA‐TyrH, additionally to L‐arabinose, 50 µM 5‐aminolevulinic acid was added. After incubation, cells were centrifuged (4000 xg, 30 min), the supernatant was discarded and the cell pellet stored at ‐20°C.

Protein purification was conducted by Ni‐NTA affinity chromatography followed with a desalting step. Cells were diluted to 10%–20% (w/v) in 50 mM potassium phosphate buffer pH 8.0 containing 20 mM imidazole and 150 mM sodium chloride (NaCl). Cells were lysed by sonication (Sonics & Materials inc, USA, 70% amplitude with 5 s on, 10 s off pulses for 10 min). The lysed cell suspension was then centrifuged at 18,500 g (4°C) for 1 h. After centrifugation, the supernatant was filtered through a syringe filter and applied to a gravity flow column containing Ni‐NTA resin (Cytiva, USA). The flowthrough was discarded and the Ni‐NTA resin was washed for 10 column volumes with 50 mM potassium phosphate buffer pH 8.0 containing 20 mM imidazole and 150 mM NaCl. After washing target protein was eluted with 50 mM potassium phosphate pH 8.0 containing 300 mM imidazole and 150 mM NaCl. The eluted protein was subsequently loaded onto a desalting column (PD‐10 desalting column, with Sephadex G‐25 resin, Cytiva, USA) and eluted with 50 mM potassium phosphate buffer pH 8.0. Purified protein was flash frozen in liquid nitrogen and stored at ‐80°C.

### 4.3 Protein Characterization

Protein concentrations were calculated using the molecular mass and molecular extinction coefficient at 280 nm of the target protein predicted via the Expasy ProtParam web tool (https://web.expasy.org/protparam). A Nanophotometer (Implen, Germany) was used for measuring the absorbance at 280 nm. Calculated parameters were: ecTnaA with His_6_‐tag: 54 927.78 Da, ε_280_ = 48,250 mM^−1^cm^−1^; avPOX with SUMO‐tag and His_6_‐tag: 78 889.02 Da, ε_280_ = 86,750 mM^−1^cm^−1^; MELA‐TyrH with SUMO‐tag and His_6_‐tag: 47 780.93 Da, ε_280_ = 42,400 mM^−1^cm^−1^.

### 4.4 Enzyme Activity Assay

Enzyme activity measurements were conducted in 200 μL reaction volume in 96‐well Flat Bottom Microplates at 25°C using a Biotek Synergy H1 microplate reader (Agilent). Reactions were started by addition of the reaction mix to the target enzyme. MELA‐TyrH activity assays were conducted similar to Carraretto et al. Standard reactions containing 50 mM KPi pH 8.0, 1 mM hydrogen peroxide, 2.5 mM indole, 5 mM MgCl_2_ and MELA‐TyrH. Absorbance was followed at 670 nm (ε_indigo_ = 4800 M^−1^ cm^−1^ at 670 nm). Effect of cascade compounds on MELA‐TyrH activity was measured similar as before by adding various hydrogen peroxide concentrations (0–10 mM) to the reaction mixture with 2.5 mM indole as substrate. Pyruvate oxidase activity was measured in a coupled assay with HRP and 2,2′‐azino‐bis(3‐ethylbenzothiazoline‐6‐sulfonic acid) (ABTS). Standard reactions containing 50 mM KPi pH 8, 5 mM pyruvate (for kinetic measurements pyruvate concentration varied up to 20 mM), 0.1 mM TPP, 5 mM MgCl_2_, 1.25 mM ABTS, 10 μg mL^−1^ HRP, and avPOX. Reactions were started by addition of enzyme to the reaction mix. Absorbance was followed at 414 nm (ε_ABTS_ = 36 mM^−1^ cm^−1^ at 414 nm [30]). Effect of cascade compounds on pyruvate oxidase activity was measured using an oxygraph instrument (Hansatech instrument Ltd, United Kingdom) with 5 or 1 mM pyruvate as a substrate in 1 mL at 25°C. A master mix was prepared including 50 mM KPi pH 8, 5 mM pyruvate (or 1 mM pyruvate), 0.1 mM TPP, 5 mM MgCl_2_, 1.25 mM ABTS, 10 μg mL^−1^ HRP, and 5 mM Indole. Reactions were started by adding the enzyme (0.05 mg mL^−1^) to the reaction mixture with constant stirring. Tryptophanase activity was measured in a coupled assay with LDH and NADH [31]. Standard reactions containing 50 mM KPi pH 8, 2.5 mM L‐tryptophan (for kinetic measurements L‐tryptophan concentration varied up to 5 mM), 5 mM MgCl_2_, 0.1 mM PLP, 0.3 mM NADH, 50 μg mL^−1^ LDH, and ecTnaA. Absorbance was followed at 340 nm (ε_NADH, 340_ = 6.22 mM^−1^ cm^−1^). Effect of cascade compounds on tryptophanase activity was measured similar as before by adding either various indole concentrations (0–5 mM) or hydrogen peroxide concentrations (0 – 10 mM) to the reaction mixture with 2.5 mM L‐tryptophan as substrate.

### 4.5 Enzyme Stability Measurements

Temperature stability (T_50_) measurements of MELA‐TyrH, avPOX and ecTnaA were conducted as in the following. 1 mg mL^−1^ of MELA‐TyrH, ecTnaA and 0.5 mg mL^−1^ of avPOX were incubate at different temperatures (20°C to 70°C) for 10 min and immediately cooled on ice for 2 min. After cooling, activity was measured as described before. Half‐life (t_1/2_) measurements of MELA‐TyrH, avPOX and ecTnaA were conducted as in the following. 1.0 mg mL^−1^ of MELA‐TyrH, ecTnaA and avPOX were incubate at 25°C. At several time points samples were taken and activity was immediately measured as described before.

### 4.6 Enzyme Cascade for Indigo Production

Test cascades for determining optimal enzyme concentrations were conducted in 200 µL volume in 2 mL Eppendorf tubes at 25°C with 600 rpm shaking. 50 mM KPi pH 8.0, 1 mM L‐tryptophan, 5 mM MgCl_2_, 0.1 mM TPP, and 0.1 mM PLP were prepared as master mix. Reactions were started with the addition of enzymes with variations in concentration of MELA‐TyrH, ecTnaA, and avPOX. Cascade samples were measured after 20 h. Reaction mixture was centrifuged for 30 min. Supernatant was analyzed as described in the following. Indigo was diluted in DMSO and measured with a Biotek Synergy H1 microplate reader (Agilent) at 620 nm against an authentic indigo standard. Test cascades for determining optimal buffer were conducted similar to the enzyme variation experiment. However, 5 mM of potassium phosphate pH 8.0 was additionally added to the reaction mixture. Before reactions were started, the storage buffer of the enzymes was switched to the buffer of choice by using spin filter. Test cascades for determining temporal enzyme addition were conducted similar to the enzyme variation experiment. Before reactions were started, either ecTnaA, ecTnaA and avPOX, or ecTnaA, and MELA‐TyrH were added to the reaction mixture. The residual enyzmes were added after 3 h of incubation. Reactions were incubated further to reach 20 h. Final enzyme cascades were conducted as follows. Cascades were prepared in 1 mL volume in a 5 mL Eppendorf tube. 50 mM KPi pH 8.0, 1 mM or 5 mM L‐tryptophan, 5 mM MgCl_2_, 0.1 mM TPP, 0.1 mM PLP, and 0.1 mM FAD were prepared as master mix. Reactions were started with the addition of enzymes in a final ratio of 0.05 U mL^−1^ MELA‐TyrH, of 0.1 U mL^−1^ ecTnaA and of 0.05 U mL^−1^ avPOX. Furthermore, similar cascades were conducted with addition of enzymes in a final ratio of 0.25 U mL^−1^ MELA‐TyrH, of 0.5 U mL^−1^ ecTnaA and of 0.25 U mL^−1^ avPOX. Reactions were conducted at 25°C at 600 rpm. Samples were taken immediately after the start, after 1, 3, 6, and 24 h.

### 4.7 Sample Preparation

For the quantification of the different compounds in the cascade, samples were taken as described in the following. At every time point, the reaction mixture was centrifuged for 10 min and 100 µL of the supernatant was added to 0.1% formic acid with 5% methanol. The residual supernatant was transferred to a new 5 mL Eppendorf tube and incubated further. The pellet from centrifugation was stored and diluted in DMSO for quantification via spectrophotometer (see following section). The methanol supernatant mixture was centrifuged again for 30 min and 20 µL were injected into the HPLC to quantify soluble compounds.

### 4.8 Compound Quantification

L‐tryptophan, indole, and 2‐oxindole were quantified via high‐performance liquid chromatography (HPLC) equipped with a UV–Vis detector (Jasco). Detection was set to 280 nm. Compound separation was conducted with a ZORBAX Eclipse XDB‐C‐18 column (4.6 x 150 mm 5‐Micron, Agilent) at 40°C using a gradient. Starting conditions are 95% eluent A (0.1% formic acid) and 5% eluent B (methanol). Starting conditions are run for 5 min isocratic and then a gradient is set from 95% A to 0% A and 5% B to 100% in 10 min. 100% B is run isocratic for 9 min and then a second gradient from 100% B to 5% B and 0% A to 95% A is run for 5 min. Afterwards 95% A and 5% B are run for 7 min isocratic to reestablish starting conditions. The flow rate is 1 mL min^−1^. Indigo quantification was based on a method previously described [32]. First, a calibration curve was prepared using commercial indigo, which was diluted in DMSO. Pelleted indigo from the enzyme cascades was diluted in DMSO and measured against a calibration curve prepared with authentic indigo. Indigo content was determined spectrophotometrically at 620 nm using a Biotek Synergy H1 microplate reader (Agilent).

## Supporting Information

Additional supporting information can be found online in the Supporting Information section.

## Funding

This study was supported by Eurostars (Project ID 3856 (OXYMEL)).

## Conflicts of Interest

The authors declare no conflicts of interest.

## Supporting information

Supplementary Material

## Data Availability

The data that support the findings of this study are available from the corresponding author upon reasonable request.
