# Supplementary material for: Biocatalytic Indigo Synthesis From L‐Tryptophan Using a Three‐Step Cascade Without Cofactor Regeneration
Source: Chembiochem. 2026 Apr 21;27(8):e70342. doi: 10.1002/cbic.70342 (PMC13096859; doi:10.1002/cbic.70342)
Supplement: Supplementary file 1 — Supplementary Material [file CBIC-27-e70342-s001.pdf]

Supporting Information

Biocatalytic Indigo Synthesis from L-Tryptophan Using a Three-Step Cascade without Cofactor Regeneration

Vivian Pascal Willers [a], Nikola Lončar [b] and Marco W. Fraaije *[a]

- **Figure S1-S8**
- **Table 1**
- **References**

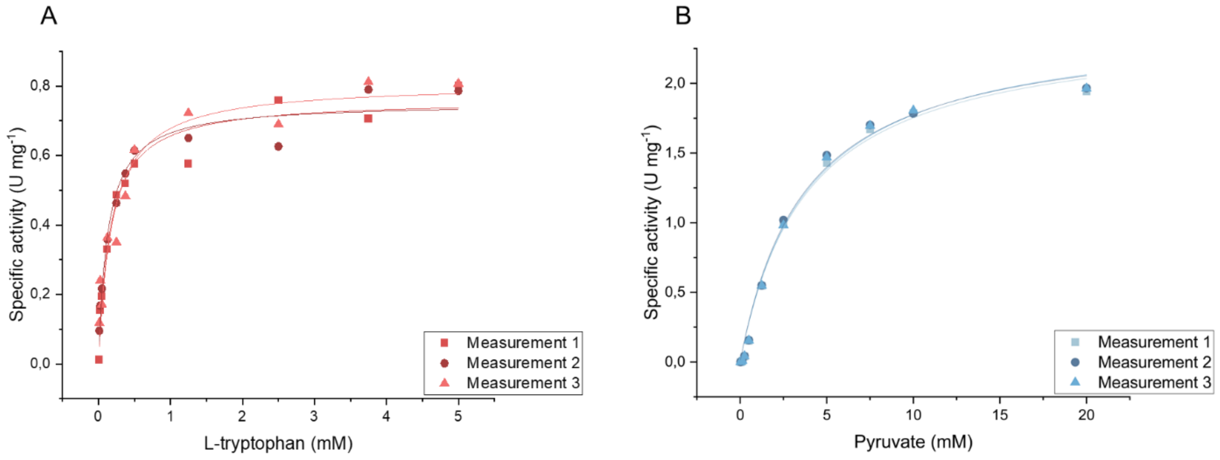

Figure S1: Kinetic measurements with Michaelis Menten fit of (A) ecTnaA and (B) avPOX.

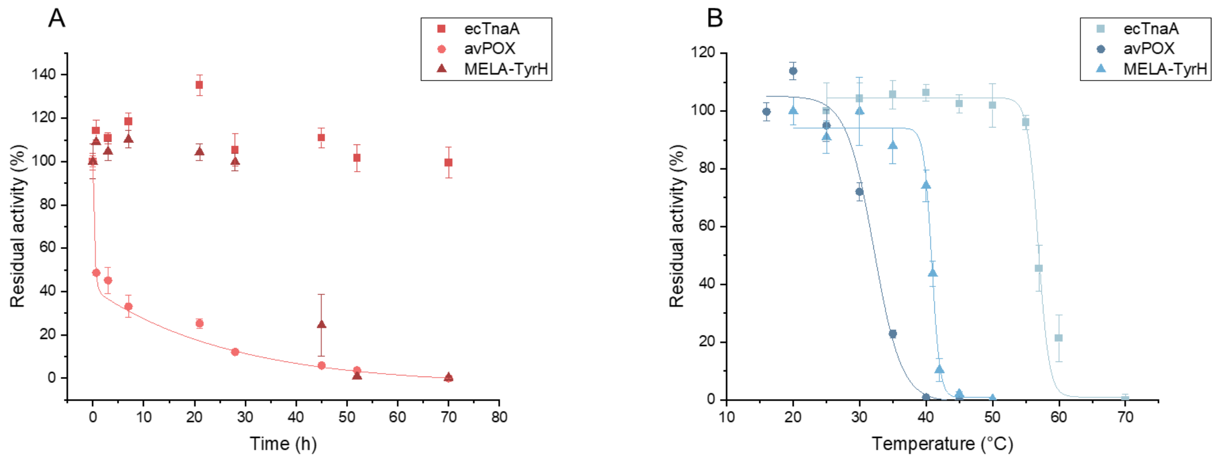

Figure S2: Stability measurements of ecTnaA, avPOX, and MELA-TyrH. (A) $t_{1/2}$ measurements with two-phase exponential decay fit for avPOX. (B) T_{50} measurements with Boltzmann fit.

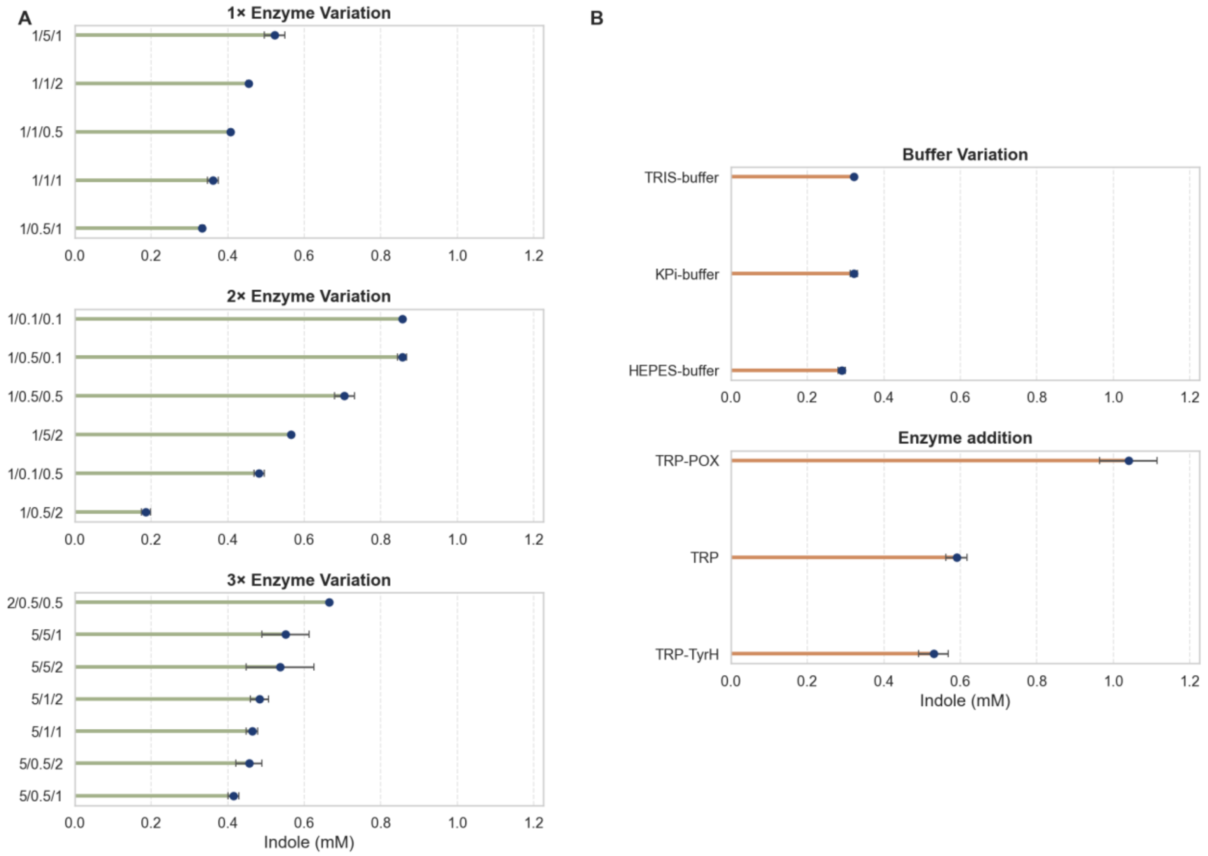

Figure S3: Initial cascade optimization with parameter variation on the y-axis and produced indole after 20 h of incubation derived from 1 mM L-tryptophan on the x-axis. (A) various enzyme variations, by increasing or decreasing the concentration of one, two or all three cascade enzymes. The order on the y-axis is always the same in position one (most far left) is ecTnaA, in position two (middle) is avPOX and in position three (right) ss MELA-TyrH (B) Additional variations of buffer (enzyme ratio (1x,0.5x,1x), as well as temporal control of enzyme addition. For the temporal control of enzyme addition the y-axis shows initial enzymes added at the start of the cascade missing enzyme(s) were added after 3 h of incubation (final ration 1x,0.5x,05x).

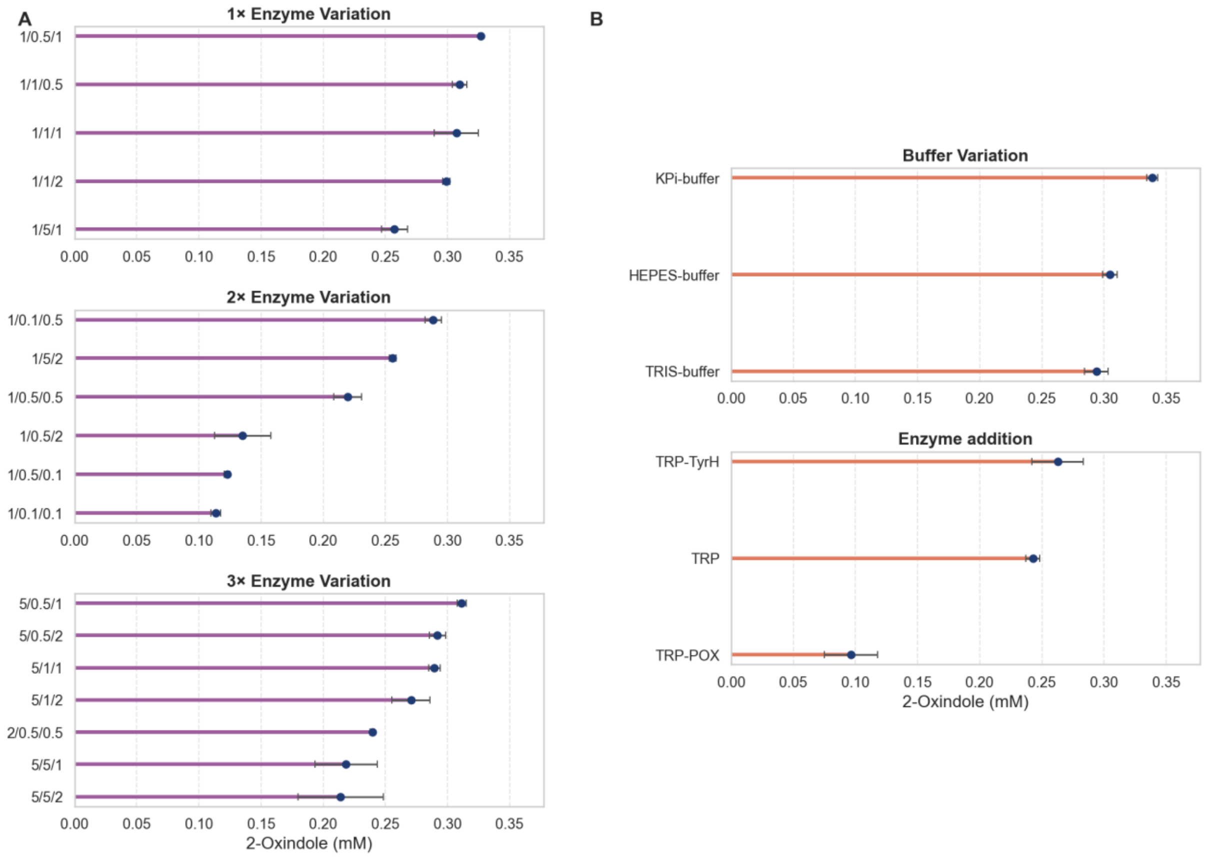

Figure S4: Initial cascade optimization with parameter variation on the y-axis and produced 2-oxindole after 20 h of incubation derived from 1 mM L-tryptophan on the x-axis. (A) various enzyme variations, by increasing or decreasing the concentration of one, two or all three cascade enzymes. The order on the y-axis is always the same in position one (most far left) is ecTnaA, in position two (middle) is avPOX and in position three (right) ss MELA-TyrH (B) Additional variations of buffer (enzyme ratio (1x,0.5x,1x), as well as temporal control of enzyme addition. For the temporal control of enzyme addition the y-axis shows initial enzymes added at the start of the cascade missing enzyme(s) were added after 3 h of incubation (final ration 1x,0.5x,05x).

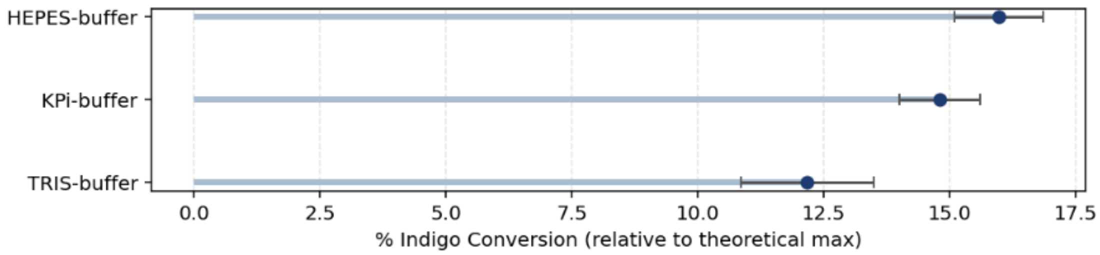

Figure S5: . Buffer variation for cascade optimization with enzyme ratio (1x / 0.5x / 1x) and produced indigo after 20 h of incubation normalized to the theoretical yield derivable from 1.0 mM L-tryptophan on the x-axis. 50 mM KPi-buffer was added to TRIS-buffer and HEPES-buffer to supply phosphate to the reaction. Enzyme order: ecTnaA / avPOX / (MELA)-TyrH.

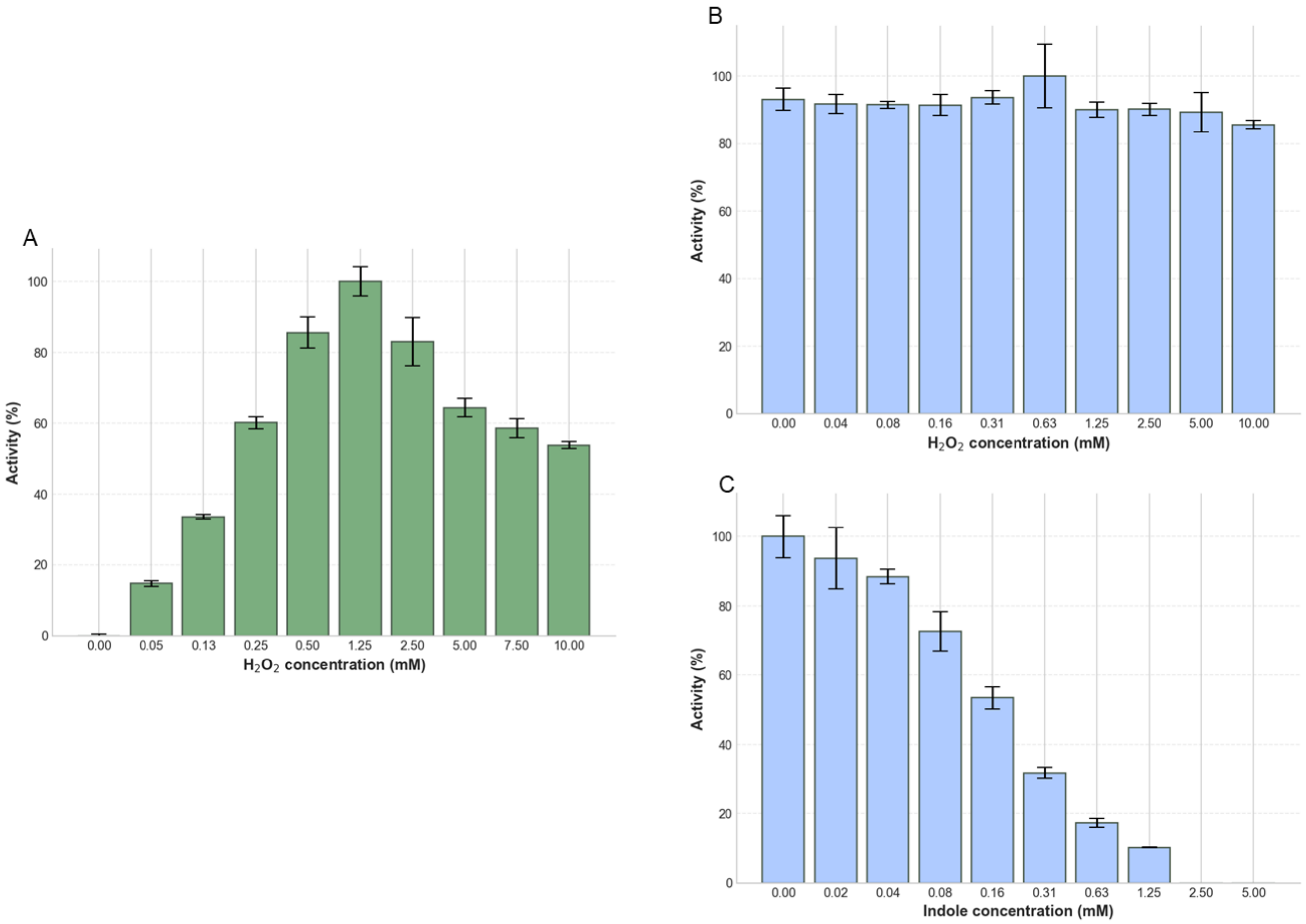

Figure S6: Activity of (A) (MELA)-TyrH on 2.5 mM indole with various concentrations of H_2O_2, (B) ecTnaA on 1 mM L-tryptophane with addition of various H_2O_2 concentrations and (C) ecTnaA on 1 mM L-tryptophan with various indole concentrations.

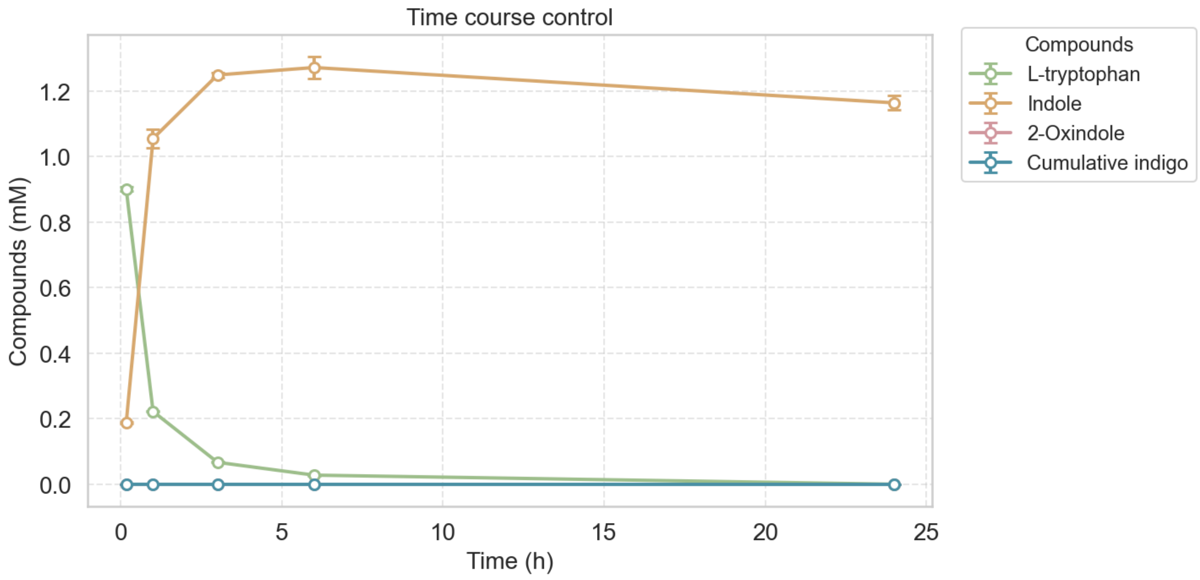

Figure S7: Time course of three enzyme cascade starting from ~1 mM L-tryptophan. Control experiment without the addition of MELA-TyrH. Time course was recorded over a time span of 24 h with timepoints taken at 0.1h, 1h, 3h, 6h and 24h.

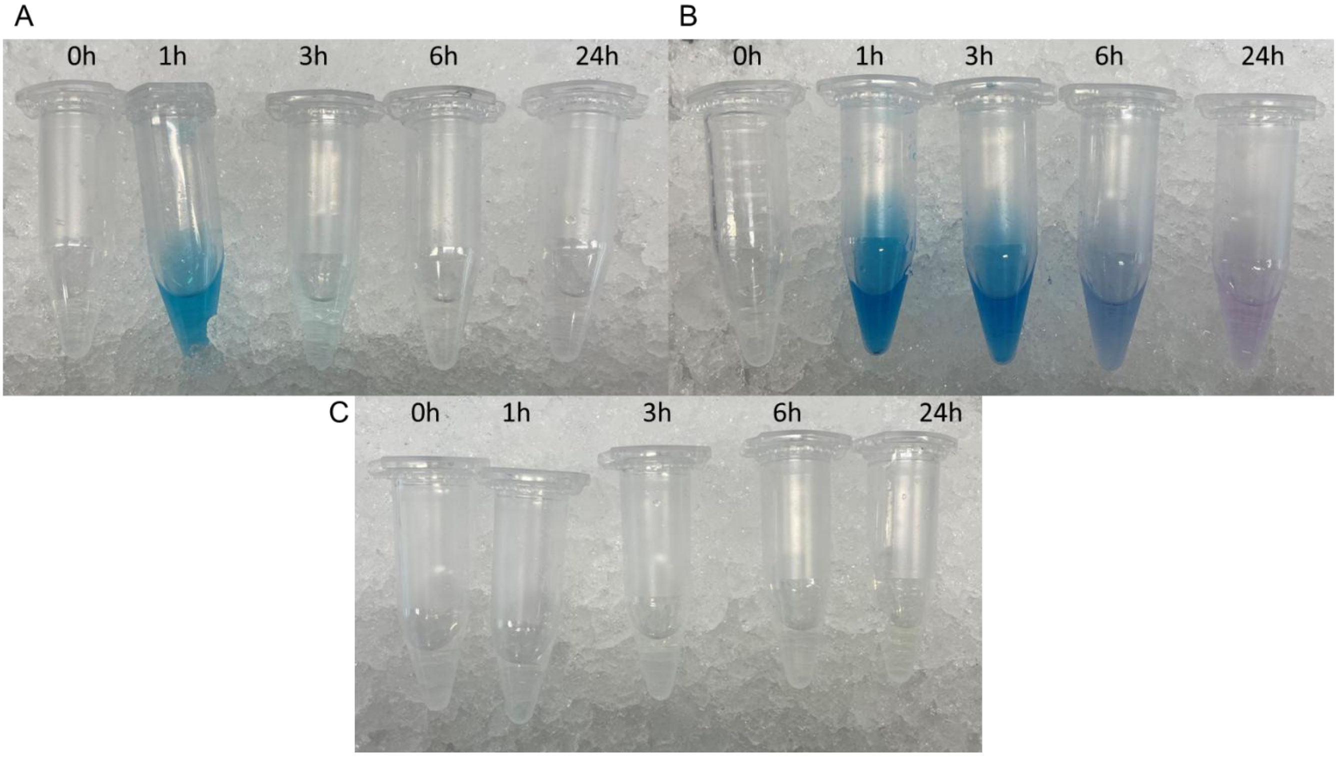

Figure S8: Cascade samples diluted in DMSO after 0h, 1h, 3h, 6h and 24h. Enzyme cascades were centrifuged, supernatant was transferred to a new five mL Eppendorf tube and precipitate was diluted in DMSO. (A) Final enzyme cascade with five times enzyme concentration with 1 mM L-tryptophan as substrate concentration. Precipitate was diluted in 1 mL DMSO. (B) Final enzyme cascade with five times enzyme concentration with 5 mM L-tryptophan as substrate concentration. Precipitate was diluted in 1 mL DMSO. (C) Control of enzyme cascade with one times enzyme concentration with 1 mM L-tryptophan as substrate concentration. Precipitate was diluted in 1 mL DMSO.

Table S1: Enzymes used in this study with information about the source Organism, Genbank ID, modifications as well as References.

Abbreviation	Organism	GenBank ID	Modification	Reference
ecTnaA	Escherichia coli	MDA4563762.1	N-His-tag	[1]
avPOX	Aerococcus viridans	AMC00721.1	N-His-tag SUMO-tag	This study
MELA-TyrH	Streptomyces sclerotialus	QXG21622.1	N-His-tag SUMO-tag	[2]

References

[1] A. N. Fabara, M. W. Fraaije, “Production of indigo through the use of a dual-function substrate and a bifunctional fusion enzyme” Enzyme Microb. Technol. **2020**, 142, 109692.

[2] D. Carraretto, L. Alonso-Cotchico, C. Martin, M. Trajković, H. L. van Beek, A. Mattevi, M. W. Fraaije, M. F. Lucas, N. Lončar, “Broadening the Catalytic Scope of the Peroxygenase Activity of a Bacterial Tyrosine Hydroxylase” ChemCatChem **2025**, 17, e202401819.
